# Using differential item functioning to evaluate potential bias in a high stakes postgraduate knowledge based assessment

**DOI:** 10.1186/s12909-018-1143-0

**Published:** 2018-04-03

**Authors:** David Hope, Karen Adamson, I. C. McManus, Liliana Chis, Andrew Elder

**Affiliations:** 1 Centre for Medical Education, The Chancellor’s Building, College of Medicine and Veterinary Medicine, The University of Edinburgh, 49 Little France Crescent, Edinburgh, Scotland EH16 4SB UK; 2 Medical Unit, St John’s Hospital, Livingston, Scotland EH54 6PP UK; 3 Research Department of Medical Education, The Medical School, University College London, Gower Street, London, WC1E 6BT UK; 4 MRCPUK Central Office, 11 St Andrew’s Place, Regent’s Park, London, NW1 4LE UK

**Keywords:** Assessment, Fairness, Bias

## Abstract

**Background:**

Fairness is a critical component of defensible assessment. Candidates should perform according to ability without influence from background characteristics such as ethnicity or sex. However, performance differs by candidate background in many assessment environments. Many potential causes of such differences exist, and examinations must be routinely analysed to ensure they do not present inappropriate progression barriers for any candidate group. By analysing the individual questions of an examination through techniques such as Differential Item Functioning (DIF), we can test whether a subset of unfair questions explains group-level differences. Such items can then be revised or removed.

**Methods:**

We used DIF to investigate fairness for 13,694 candidates sitting a major international summative postgraduate examination in internal medicine. We compared (a) ethnically white UK graduates against ethnically non-white UK graduates and (b) male UK graduates against female UK graduates. DIF was used to test 2773 questions across 14 sittings.

**Results:**

Across 2773 questions eight (0.29%) showed notable DIF after correcting for multiple comparisons: seven medium effects and one large effect. Blinded analysis of these questions by a panel of clinician assessors identified no plausible explanations for the differences. These questions were removed from the question bank and we present them here to share knowledge of questions with DIF. These questions did not significantly impact the overall performance of the cohort. Group-level differences in performance between the groups we studied in this examination cannot be explained by a subset of unfair questions.

**Conclusions:**

DIF helps explore fairness in assessment at the question level. This is especially important in high-stakes assessment where a small number of unfair questions may adversely impact the passing rates of some groups. However, very few questions exhibited notable DIF so differences in passing rates for the groups we studied cannot be explained by unfairness at the question level.

**Electronic supplementary material:**

The online version of this article (10.1186/s12909-018-1143-0) contains supplementary material, which is available to authorized users.

## Background

Promoting fairness in assessment is an important priority. Entrance to medical school and progression as a doctor should be determined by ability, not ethnicity or sex [1]. Recognising this, many governing bodies now explicitly require educational processes to be fair in respect to “protected characteristics” such as ethnicity and sex [2]. The need for fairness is now formally identified in key reference guides on test construction, whereby unfairness is seen as irrelevant variance threatening the validity of the construct, which must therefore be identified and removed. [3] Developing assessment practices that differentiate candidates by ability without acting in a discriminatory way in relation to protected characteristics is a particular necessity for those running professional assessments [4].

Despite such aspirations, protected characteristics remain influential predictors of outcome at every stage of medical training. A meta-analysis of 22 studies found moderate (*d* = 0.42) effects whereby ethnically non-white UK graduates underperformed relative to white UK graduates in assessments at both undergraduate and postgraduate levels. Nor is it merely an assessment issue – questionnaires evaluating the postgraduate working experience find ethnically non-white trainees experience lower levels of satisfaction [5]. In relation to sex, there is evidence that men and women prefer different types of assessment and in at least one case – true/false MCQs (Multiple Choice Questions) where candidates are penalised for incorrect answers – the format of the assessment can produce significant sex differences [6].

The overall underperformance of candidates from a particular group is generally referred to as *Differential Attainment* (DA), and it can have multiple and complex origins – as seen for instance in ethnic and sex differences in school level attainment in UK students [7]. In postgraduate assessment concerns over potential bias have often focused on clinical examinations where examiners are in direct contact with the candidate. DA relating to ethnicity and sex have been identified in many postgraduate examinations, but examiner bias has not been found to explain these differences in, for instance, either MRCP(UK) [8, 9] or MRCGP [10]. Despite significant professional and public interest and a judicial review the causes of such differences remain unclear, and the possibility that they relate to as yet unidentified characteristics of the candidates or their training experience and environment, rather than identifiable characteristics of the examinations, is under active exploration [9, 11, 12]. Proposed explanations have included subconscious bias, varying standards at different educational facilities, or experts favouring those similar to themselves [11, 12]. These explanations are speculative or, in some cases, have not been borne out by research, but all sources we are familiar with accept that differential attainment is not due to inherent ethnic or sex differences [11].

Differential attainment also exists in written postgraduate assessments [9, 13], further suggesting that factors other than the influence of direct examiner bias are in operation, not the least since most of these assessments are comprised of machine marked MCQ questions. Again, however, the precise reasons for the differences are not fully understood. A possible contributor may be the relative degree of difficulty of specific questions or groups of questions for candidates with different characteristics. A simple example may be that candidates who practice in a country where a given condition is rarely encountered, may have more difficulty with questions regarding the condition. However, even in situations where candidates have received their primary medical qualification in the same country, in this case the UK, differences in overall performance in examinations between candidates with different protected characteristics (e.g. sex, ethnicity) can be identified, and the possibility that this could relate to inadvertent differential item functioning explored.

The identification of differential attainment in many types of assessment and levels of training supports the absence of a single, readily identifiable root cause and implies a multifactorial problem [13]. A key responsibility of all providers of high stakes assessments is to seek and identify any sources of bias in their examination, and to evaluate the cause, extent and implication of any bias that is identified. Solutions can then be proposed on a case-by-case basis. As one part of this process, MCQs must be carefully evaluated to explore whether particular specialties, topics, or question structures exhibit bias [14, 15]. By doing this we can better understand the causes of bias in written assessment and prevent such bias in the future.

Simple tests for bias may be uninformative. A comparison of mean scores (e.g. “on question 7, do males perform differently from females?”) provides a useful diagnostic but only compares average performance level in different groups. This may conceal performance differences at the lower end of the ability curve (important as such candidates are at risk of failure) and the upper end (where candidates are in contention for merits and awards).

**Fig. 1:**
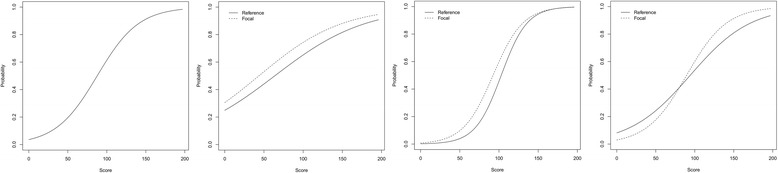
A comparison of Differential Item Functioning Curves. Note: “Score” is the overall performance on the examination. “Probability” is the likelihood of the examinee answering this question correctly. From left to right, the plots show no DIF, uniform DIF, non-uniform DIF and crossing DIF. Where there are two curves, the gap between the curves shows the DIF effect

A more robust method for evaluating item-level bias is to test for *Differential Item Functioning* (DIF), which takes into account differences at every level of candidate ability [16, 17]. Tests for DIF compare two (or more) groups of interest: whites vs. non-whites, for instance, or males vs. females. The test compares performance for these groups on each test question at every stage of the overall ability curve. It therefore allows exploration of whether bias is concentrated in particular areas (borderline or excellent candidates) and how the bias interacts with ability. DIF can define *no bias*, *uniform* differences (group A outperforms group B across the ability curve), *non-uniform* differences (group A outperforms group B, but the size of the gap varies), or even *crossing* differences (group A outperforms group B on some parts of the curve, but the opposite is true at other parts of the curve). Figure 1 illustrates an example. After identifying questions with these characteristics, test setters and question writers can then review or remove those questions from future forms of the examination.

DIF analysis can also permit the assessment of the impact of removing questions with DIF, using questions without DIF as anchor points to explore new questions’ characteristics and reporting the magnitude of question bias in a convenient way [17]. Importantly, it can provide reassurance that DIF is limited or absent in an assessment by estimating the difference between the actual level of DIF items against that which would be expected by chance alone.

In summary, tests for DIF can accurately detect potential bias across the ability curve at the question level and identify problem questions for evaluation or revision. Although many free online tools exist to test for DIF and can be used by any interested party [18, 19] they have yet to be widely applied in medical education or the academic governance of high stakes postgraduate examinations in the UK.

In this study, we investigated the performance of 13,694 candidates sitting the [MRCP(UK) Part 1 examination], a high stakes postgraduate assessment in internal medicine and we compare (a) ethnically white UK graduates against ethnically non-white UK graduates and (b) male UK graduates against female UK graduates. DIF was used to test 2773 questions across 14 sittings, each initially comprising two three hour papers of 100 questions each. We report the results of the DIF analysis alongside a review of questions exhibiting DIF.

## Methods

### Participants

All analyses were carried out on different diets (forms) of the same high stakes postgraduate examination. The examination is a criterion-referenced written examination comprised of 200 questions, sat in two separate 3 hour papers of 100 questions each, designed to assess core clinical knowledge. Blueprinting, item content and standard setting are all developed and determined by panels of experts with relevant clinical backgrounds.

Fourteen sittings were included in this analysis: three each for 2011, 2012, 2013 and 2014, and two for 2015. In total, there were 47,048 candidate entries across the fourteen sittings, which included candidates who had not qualified in the UK. Candidates had been invited to provide information on their protected characteristics, which included sex, whether their primary medical qualification was from the UK or not and whether they were ethnically white or ethnically non-white, as part of the routine application process for the examinations. 6047 candidates failed to provide information on any or all of these questions, and a further 27,307 sittings were excluded due to the candidate not having a UK PMQ. This left 13,694 candidate sittings – all UK graduates – in the present study with graduates who were resitting being included as separate entries for the purpose of the present study. Consequently, some candidates had multiple entries in the analysis, with each entry being a single attempt at the examination, sat during different diets. No candidate had multiple entries for any individual diet. Candidates routinely provided consent for anonymised analysis of examination results, and approval was granted by the MRCP(UK) board for the work to be undertaken.

### Statistical analyses

All analyses were carried out in *R* [20]. For step-by-step guides on how to replicate the analyses described here see the guidance notes provided in the package *difR* [19] or for an alternative see the *lordif* package [18]. We ran a DIF analysis separately on each of the 14 datasets. Initially, there were 200 questions in each sitting. Post-hoc analysis of question quality (including discrimination and facility) led to 27 questions being discounted over the 14 sittings. These were not included in the DIF analysis. The passing standard for each diet was determined through statistical equating, a process designed to ensure comparable standards across different diets [21]. The DIF analysis used the probability of getting the item correct as the dependent variable.

For each question we calculated DIF using the logistic regression method [16] which can detect uniform and non-uniform effects, and significance was evaluated via the Wald test. Sex and ethnicity were modelled separately. In total, 2773 tests were run for sex and 2773 tests were run for ethnicity for a total of 5546 tests: one test per question per characteristic of interest. Inevitably this raises the prospect of a significant number of false positives and DIF procedures routinely correct for this possibility. With no corrections, 139 “significant” results would be expected for sex and 139 for ethnicity: a total of 278 “significant” results in a random dataset with no genuine DIF results [22, 23]. In our DIF procedure we adjusted the alpha level to .01 and report on tests significant at that level as a compromise between being too stringent (and so missing signs of DIF) and too lenient (reporting DIF where none occurs). The choice between being too lenient and too stringent when making statistical adjustments is to a great extent a matter of judgment. We note however that a strict Bonferroni correction – which fully adjusts for the problem of multiple comparisons – for the conventional *p* < .05 would, with 5546 tests, require p < .05/5546 = 9.01 × 10^− 6^ for significance after adjusting for multiple testing.

We report the number of questions identified as exhibiting DIF. We further investigated any questions with either a medium or large effect size as measured by Nagelkerke’s R^2^ [19]. All eight such questions were passed to a ten-member panel of clinicians with varying types of involvement with postgraduate examinations (question writers, standard setters, clinical examiners) who were informed only that the questions exhibited DIF, but not the nature of the DIF effect, its strength, or which group was disadvantaged.

## Results

Table 1 shows the distribution of DIF questions in the diets studied, and their effect and direction according to sex and ethnicity. In total, 217 questions exhibited DIF using the .01 alpha level (as opposed to an expected 645 to occur by chance using a .05 alpha level) but the effects were almost always negligible: that is, the DIF has only a very small impact on group-level differences [19]. The frequency of DIF questions per paper varied over time using the .01 alpha level, from a minimum of five to a maximum of 42. Eight medium or large DIF effects were identified, and received further analysis. For comparison, using a too-liberal procedure that did not correct for multiple comparisons yielded a total of 645 significant results. None of the nominally significant effects reached a Bonferroni corrected level of 9.01 × 10^− 6^.

**Table 1:** A summary of items with Differential Item Functioning

Diet	Total	Ethnicity			Sex			Both		
		Negligible	Medium	Large	Negligible	Medium	Large	Negligible	Medium	Large
2011 (1)	10	6			4					
2011 (2)	5	5								
2011 (3)	33	20			10			3		
2012 (1)	16	9	1		5			1		
2012 (2)	8	3			5					
2012 (3)	26	3			22	1				
2013 (1)	8	3			4	1				
2013 (2)	10	5			5					
2013 (3)	15	5	1		8			1		
2014 (1)	22	7			12			3		
2014 (2)	6	4			2					
2014 (3)	42	10			29	2	1			
2015 (1)	6	5			1					
2015 (2)	10	2			7	1				
Combined	217	87	2	0	114	5	1	8	0	0

Note: Following the convention on effect sizes for DIF described by Magis et al., effect sizes below the medium threshold are classed as negligible rather than small. Blanks indicate no items of that magnitude. “Total” refers to total number of significant DIF tests. As some items exhibited DIF for both ethnicity and sex, the total number of *items* identified as problematic is slightly lower

Table 2 reports the detailed breakdown of the eight notable DIF cases that had either medium or large effect sizes, provides a visual overview of the DIF effect, the question text and the options. In summary, most of the questions showed DIF with regards to sex, not ethnicity. All types of DIF (uniform, non-uniform and crossing) were found. For the two questions showing notable DIF with regards to ethnicity, white UK graduates were advantaged in both cases. One item exhibited a uniform curve and one a crossing curve. For the six questions showing notable DIF with regards to sex, the picture was mixed but female graduates tended to be advantaged over male graduates. Two items exhibited uniform curves, two non-uniform curves and two crossing curves.

**Table 2:**
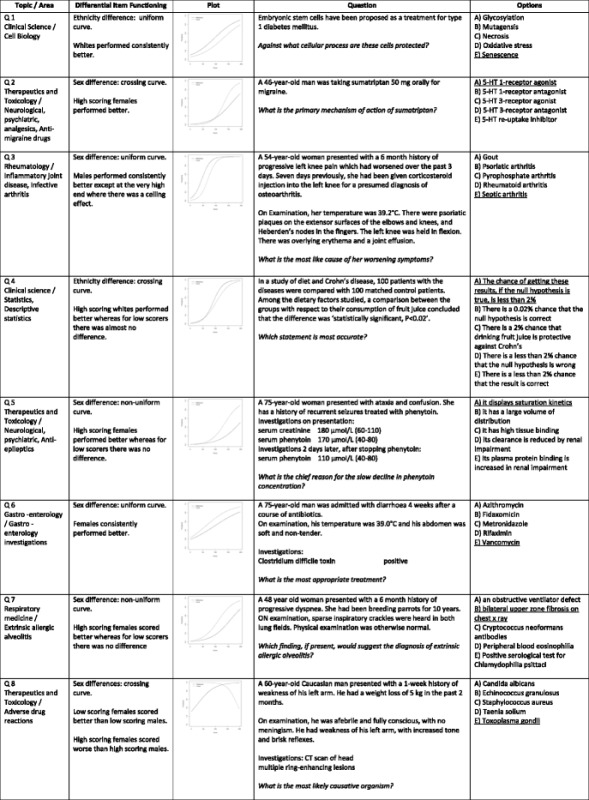
Eight items exhibiting medium- or large- effects of Differential Item Functioning

Note: “Topic/Area” summarises the subject matter. “Differential Item Functioning” describes whether there was a sex or ethnicity difference, the type of curve observed, and gives a description of the trend. For “plot,” probability indicates the probability of answering the item correctly. Score indicates the candidate’s performance overall. Typically, candidates who score well on the exam should be more likely to answer the item correctly, but this is not always the case. The solid “reference” line refers to males, or ethnically white UK graduates, depending on whether a sex or ethnicity difference was found. The dashed line refers to females or ethnically non-white UK graduates. See Fig. 1 for more details about curve types. “Question” is the text of the question as seen by candidates. “Options” lists the five available options. The keyed answer is underlined

[Note for publication – high resolution versions of each plot are included as “Additional file 1”]

### Expert review

Following this analysis, the eight questions were reviewed by an expert panel comprised of ten clinicians of diverse backgrounds (UK trained and non-UK trained, male and female, ethnically white and ethnically non-white) to evaluate the possible causes of DIF. The analysis was blinded. Reviewers were not able to consult with other panel members, and knew only that DIF was present – not the type or magnitude. No reviewer was able to suggest any cogent explanation for the DIF for any item.

## Discussion

This paper presents the first evaluation of DIF in the context of a large postgraduate medical examination, the MRCP(UK). In this dataset, which relates to a single high stakes postgraduate knowledge based assessment of single best answer MCQ design, and is comprised of an extremely large sample size of test takers and questions, DIF was detected using simple software and statistical tools. Importantly however the analysis shows most such effects are negligible and only a very small number of questions exhibit DIF of a medium or large effect size. Furthermore, no clear cause of the observed DIF can be derived from analysis of independent blinded panel review.

This research shows that methods to evaluate DIF successfully used in other fields can be applied to medical education [16, 17]. Differential attainment in postgraduate examinations cannot primarily be explained by bias at the question level.

We believe that this analytical strategy is simple, advantageous and suggests that DIF evaluation can be one of a range of measures to test fairness. Routine analysis will identify the – apparently rare – cases of notable DIF and so promote fairness at the borderline of pass/fail decisions. Connecting such psychometric analyses with expertise in question writing allows for the development of better training in question-writing, promotes awareness of the possibility of bias in written assessment, and allows for the development of a body of knowledge around questions most likely to produce bias. The notable DIF questions have all been withdrawn from the question bank and publicised amongst question writers, even though the reason for their differential functioning is unclear. As more items are identified in future analyses it may be that common structural or content issues will emerge as explanations.

The study had some limitations. Replications on novel datasets are required to evaluate how common DIF effects are in other areas of assessment. As some candidates are prone to resitting, and therefore will have appeared in multiple datasets, exploring whether resitting candidates exhibit DIF compared to non-resitters of similar ability levels would be useful in extending this analysis. Furthermore, evaluation at the undergraduate level would be very helpful given the importance of differential attainment in application to medical school and performance at medical school. While we were able to evaluate possible reasons for question bias, we consulted experts, rather than novices, and work with candidates applying to sit assessment would be a useful comparator to identify why questions might (or might not) be considered biased. Exploring other features of the items – such as length, item type, language complexity and so on – may be a useful avenue of exploration. Furthermore, Identifying and publicising problem questions could be a powerful tool for advancing fairness if other organisations repeated this analysis and pooled the results. Importantly, the causes of DIF were unexplained and whether they are false positives or due to some yet unstudied factor remains open. Relatedly, this method was only one of a family of DIF procedures, and comparing it against other techniques (e.g. Mantel-Haenszel) may yield slightly different results.

This research has provided a model for the monitoring and removal of questions with DIF in high stakes assessment. By making such analyses routine, we can promote fairness at the question level and ensure that assessment is based solely on candidate ability. Questions showing DIF were rare, and there was usually little clear relationship of the substantive content of the questions to the DIF found, suggesting that in most cases the DIF was of little meaning in relation to the overall performance of the candidates.

Our expert review was blinded to the nature or direction of DIF, but the reviewers knew that DIF was present. A more exacting test, perhaps to be carried out in the future, would be to give examiners a balanced set of questions and say that a third showed DIF evidence of sex differences, a third showed DIF evidence of ethnicity differences, and a third showed no evidence of DIF by sex or ethnicity, and ask them to judge which question was in each type. Our feeling is that examiners probably would be unable to do that task correctly, suggesting that there are few obvious features about these questions which are related to the presence of DIF.

## Conclusions

Overall it seems highly unlikely that any candidates were disadvantaged by the small effects that were found. The differential attainment of candidates in the various groups does however remain, and remains unexplained, but cannot be attributed to differential item functioning in relation to sex or ethnicity of particular questions in this multiple choice assessment across the range of diets analysed.

## Additional file

Additional file 1: High resolution images of the plots contained in the paper. (ZIP 2058 kb)

## Abbreviations

DA: Differential Attainment
DIF: Differential Item Functioning
MCQ: Multiple Choice Question
MRCGP: Membership of the Royal College of General Practitioners
MRCP(UK): Membership of the Royal Colleges of Physicians (United Kingdom)
PMQ: Primary Medical Qualification

## References

[1] Tsouroufli M, Malcolm I. Equality, diversity and fairness in medical education: international perspectives. Med Educ. 2015;49:4–6. 10.1111/medu.12601. 10.1111/medu.12601 25545562

[2] General Medical Council (2009). Tomorrow’s doctors.

[3] American Educational Research Association, American Psychological Association, National, Council on Measurement in Education, Joint Committee on Standards for Educational and, Psychological Testing (U.S.) (2014). Standards for educational and psychological testing.

[4] Eva KW (2015). Moving beyond childish notions of fair and equitable. Med Educ.

[5] Gill D (2016). The association between trainee demographic factors and self-reported experience: analysis of general medical council National Training Survey 2014 and 2015 data. JRSM Open.

[6] Kelly S, Dennick R (2009). Evidence of gender bias in true-false-abstain medical examinations. BMC Med Educ.

[7] McManus IC, Woolf K, Dacre J (2008). The educational background and qualifications of UK medical students from ethnic minorities. BMC Med Educ.

[8] McManus IC, Elder AT, Dacre J (2013). Investigating possible ethnicity and sex bias in clinical examiners: an analysis of data from the MRCP(UK) PACES and nPACES examinations. BMC Med Educ.

[9] Dewhurst N, McManus C, Mollon J, Dacre J, Vale A (2007). Performance in the MRCP(UK) examination 2003-4: analysis of pass rates of UK graduates in relation to self-declared ethnicity and gender. BMC Med.

[10] Wakeford R, Farooqi A, Rashid A, Southgate L (1992). Does the MRCGP examination discriminate against Asian doctors?. BMJ.

[11] Denney ML, Freeman A, Wakeford R (2013). MRCGP CSA: are the examiners biased, favouring their own by sex, ethnicity, and degree source?. Br J Gen Pr.

[12] The Queen on the application of Bapio Action Ltd [Cliamant] v Royal College of General Practitioners [First Defendant] and General Medical Council [Second Defendant], in the High Court of Justice, Queen’s Bench Division, The Administrative Court. 10th April 2014. EWHC 1416 (Admin) 2014, Available at http://www.rcgp.org.uk/news/2014/may/~/media/Files/News/Judicial-Review-Judgment-14-April-2014.ashx. n.d.

[13] Woolf K, Potts HWW, McManus IC (2011). Ethnicity and academic performance in UK trained doctors and medical students: systematic review and meta-analysis. BMJ.

[14] McCoubrie P. Improving the fairness of multiple-choice questions: a literature review. Med Teach. 2004;26:709–12. 10.1080/01421590400013495. 10.1080/01421590400013495 15763874

[15] Downing SM (2003). Validity: on the meaningful interpretation of assessment data. Med Educ.

[16] Swaminathan H, Rogers HJ (1990). Detecting differential item functioning using logistic regression procedures. J Educ Meas.

[17] Clauser BE, Mazor KM (1998). Using statistical procedures to identify differentially functioning test items. Educ Meas Issues Pract.

[18] Choi SW, Gibbons LE, Crane PK (2011). Lordif: an R package for detecting differential item functioning using iterative hybrid ordinal logistic regression/item response theory and Monte Carlo simulations. J Stat Softw.

[19] Magis D, Béland S, Tuerlinckx F, Boeck PD (2010). A general framework and an R package for the detection of dichotomous differential item functioning. Behav Res Methods.

[20] Ihaka R, Gentleman R (1996). R: a language for data analysis and graphics. J Comput Graph Stat.

[21] McManus IC, Chis L, Fox R, Waller D, Tang P (2014). Implementing statistical equating for MRCP (UK) parts 1 and 2. BMC Med Educ.

[22] Abdi H (2007). The Bonferonni and Šidák corrections for multiple comparisons. Encycl Meas Stat.

[23] Dunn OJ (1961). Multiple comparisons among means. J Am Stat Assoc.

