# Supplementary figures and images for: Using differential item functioning to evaluate potential bias in a high stakes postgraduate knowledge based assessment
Source: BMC Med Educ. 2018 Apr 3;18:64. doi: 10.1186/s12909-018-1143-0 (PMC5883583; doi:10.1186/s12909-018-1143-0)

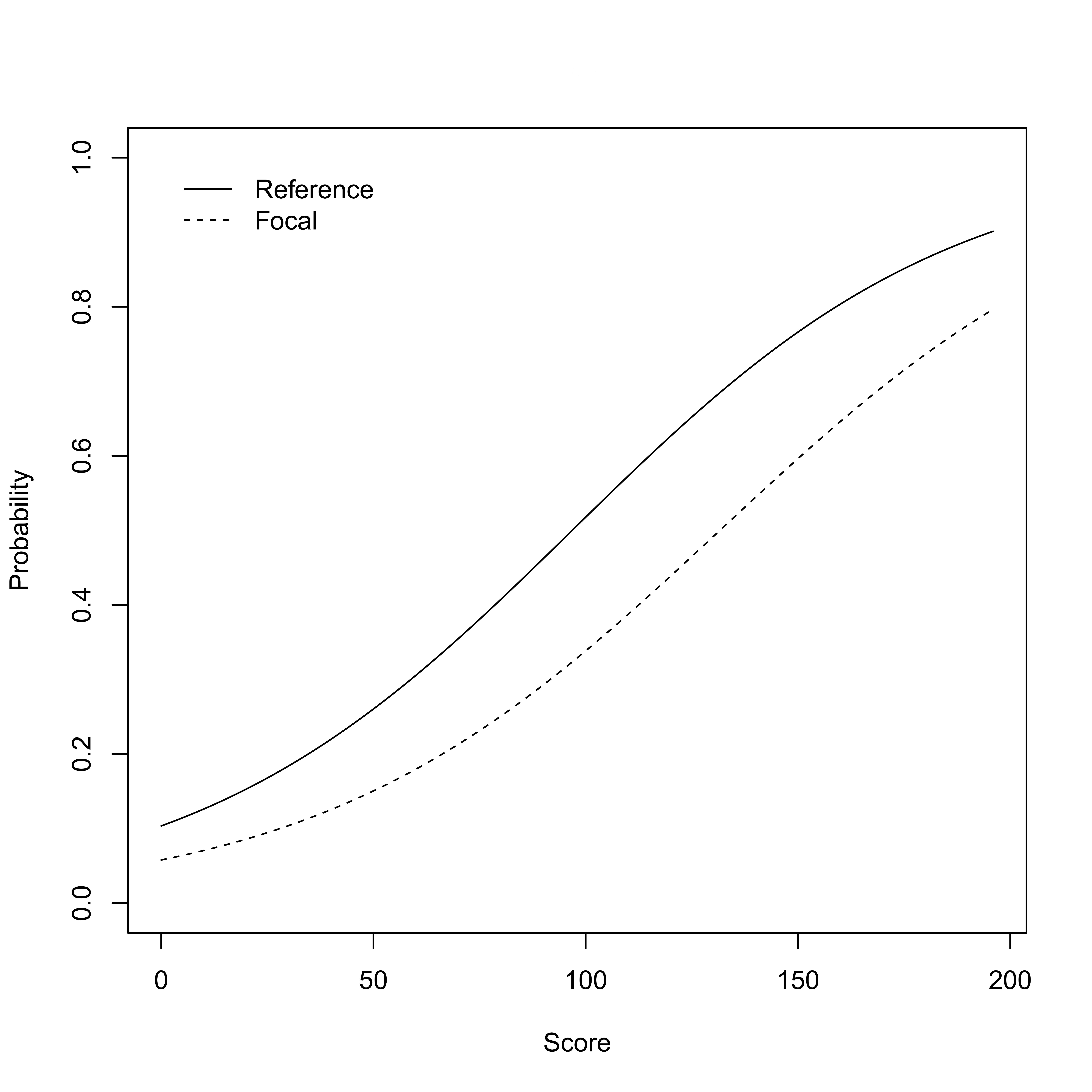

Supplement: Supplementary file 1 — High resolution images of the plots contained in the paper. (ZIP 2058 kb) [file 12909_2018_1143_MOESM1_ESM.zip › Q1FINALR2.jpg]

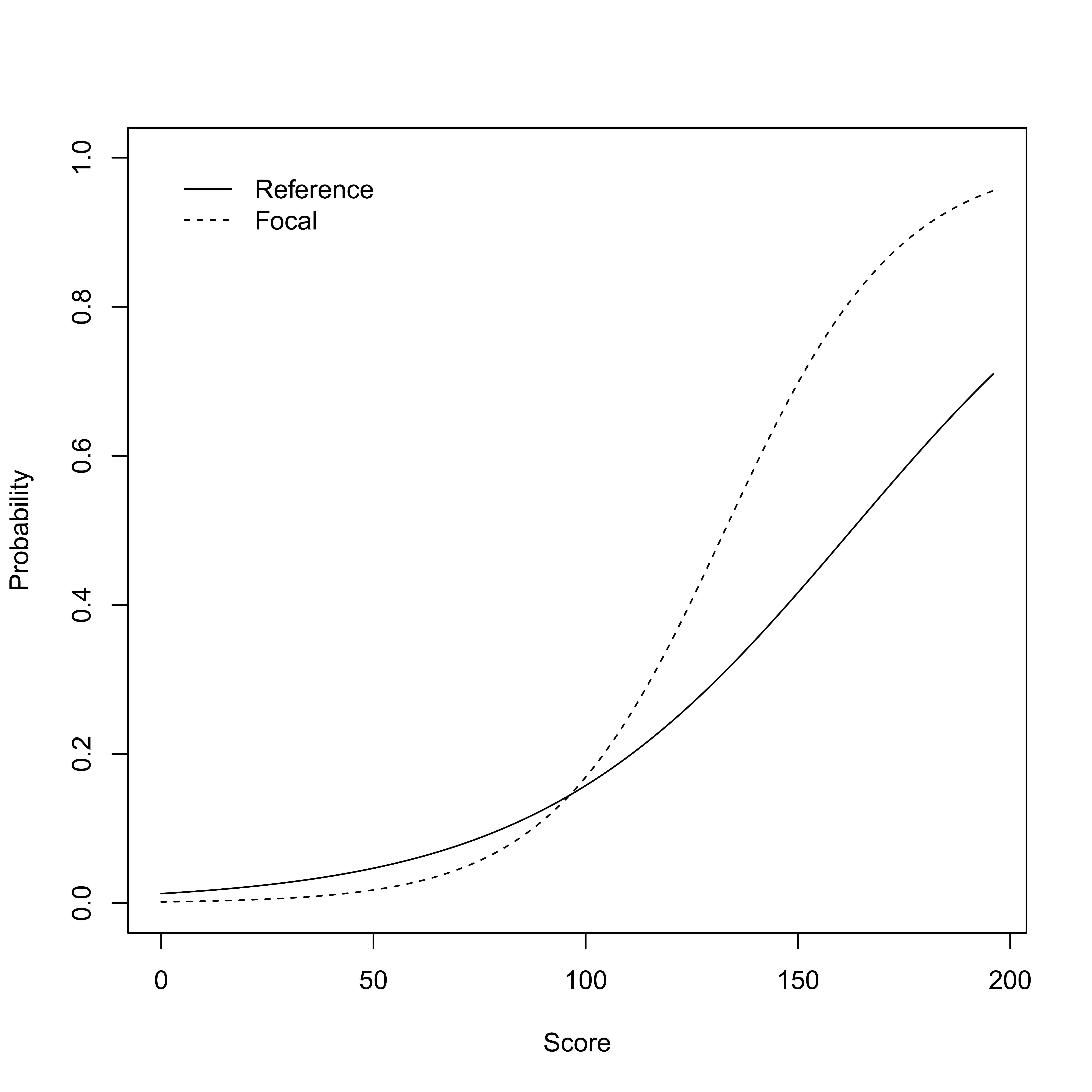

Supplement: Supplementary file 1 — High resolution images of the plots contained in the paper. (ZIP 2058 kb) [file 12909_2018_1143_MOESM1_ESM.zip › Q2FINALR2.jpg]

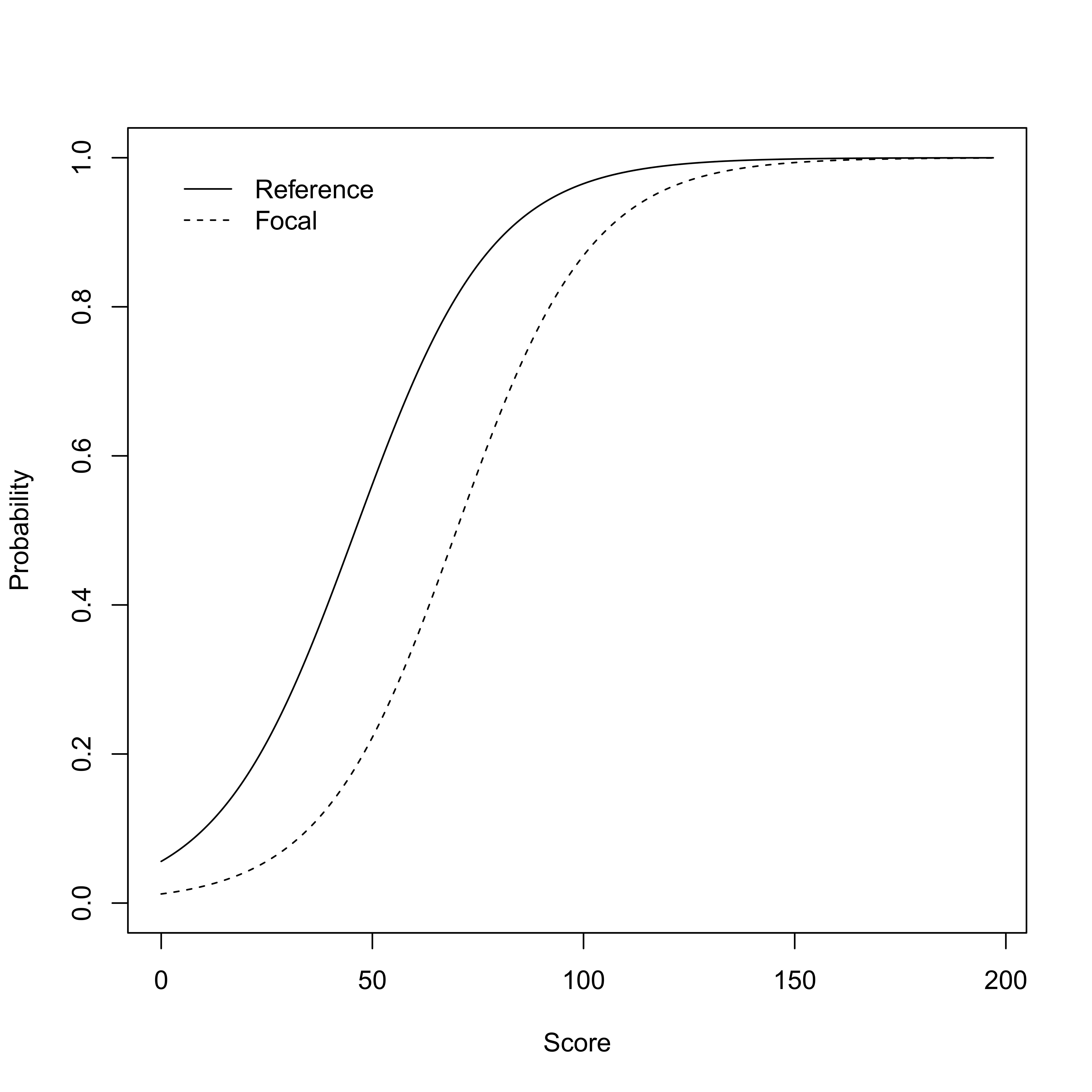

Supplement: Supplementary file 1 — High resolution images of the plots contained in the paper. (ZIP 2058 kb) [file 12909_2018_1143_MOESM1_ESM.zip › Q3FINALR2.jpg]

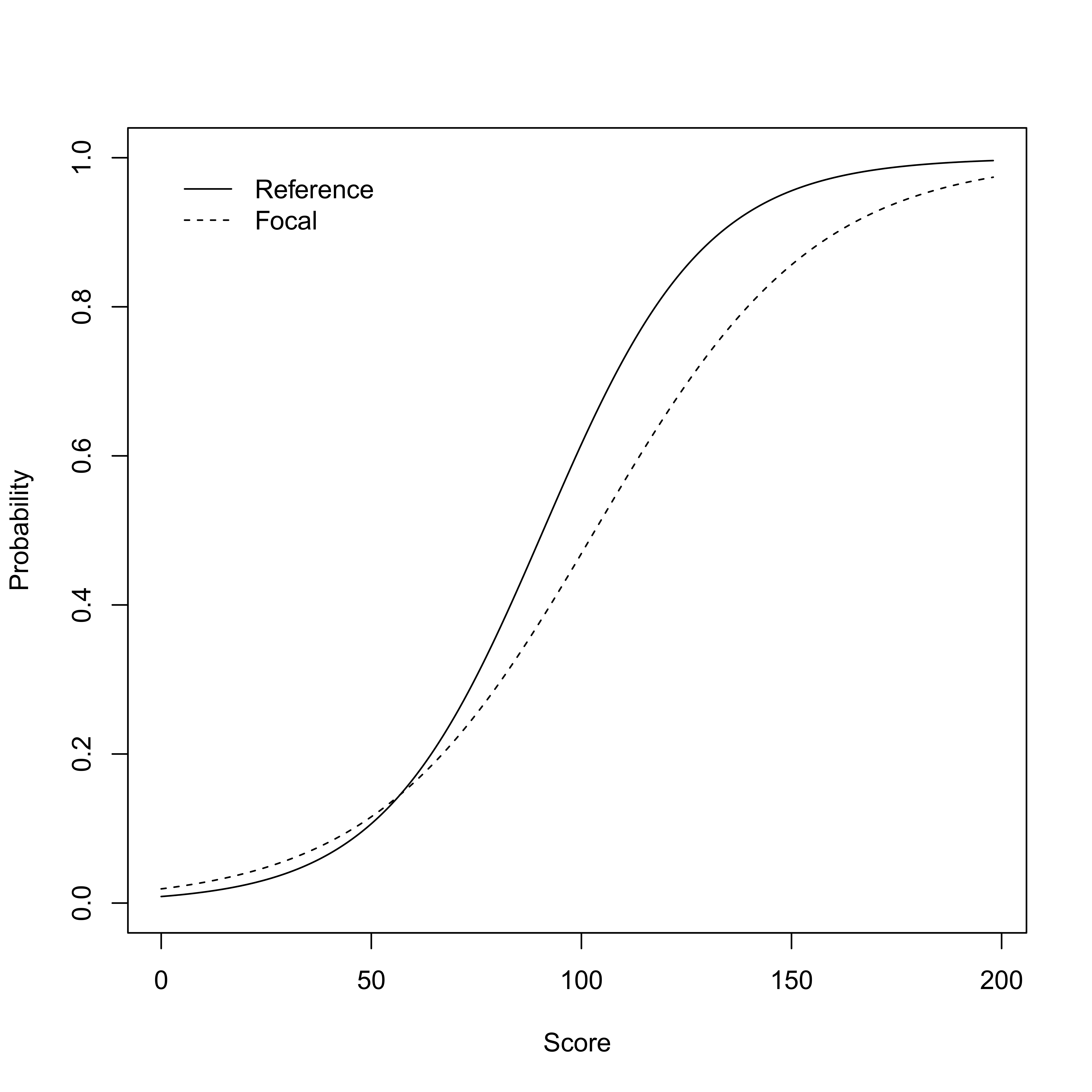

Supplement: Supplementary file 1 — High resolution images of the plots contained in the paper. (ZIP 2058 kb) [file 12909_2018_1143_MOESM1_ESM.zip › Q4FINALR2.jpg]

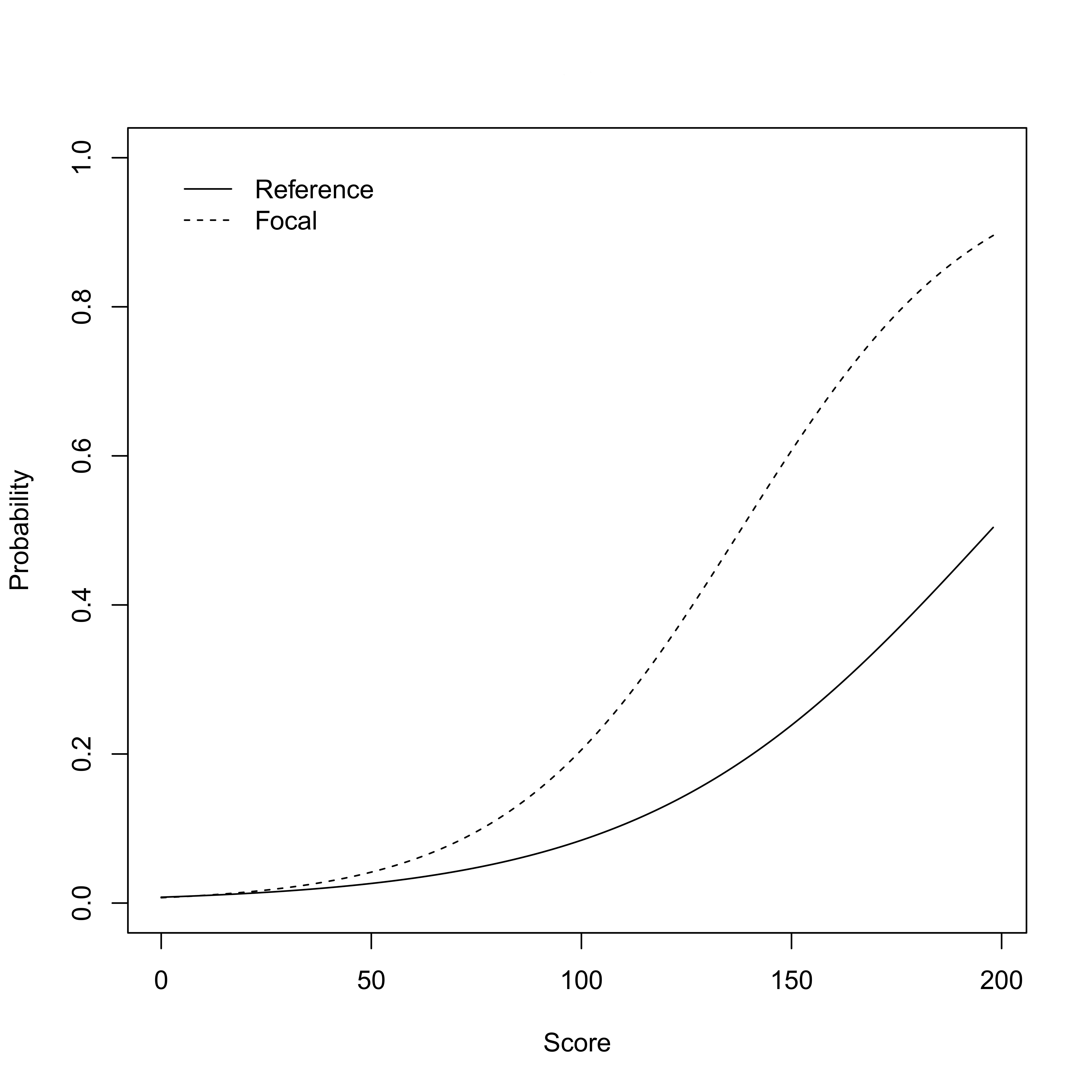

Supplement: Supplementary file 1 — High resolution images of the plots contained in the paper. (ZIP 2058 kb) [file 12909_2018_1143_MOESM1_ESM.zip › Q5FINALR2.jpg]

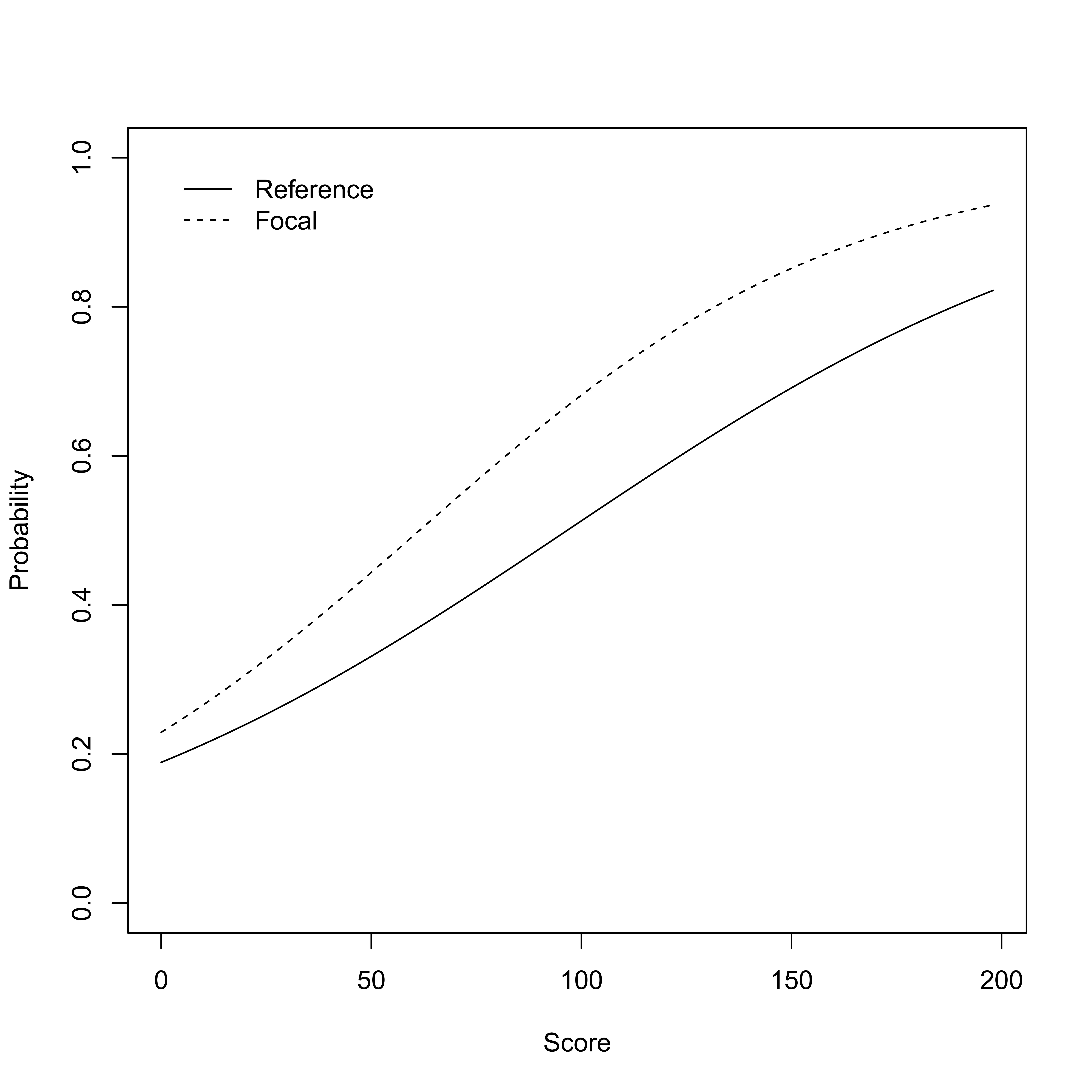

Supplement: Supplementary file 1 — High resolution images of the plots contained in the paper. (ZIP 2058 kb) [file 12909_2018_1143_MOESM1_ESM.zip › Q6FINALR2.jpg]

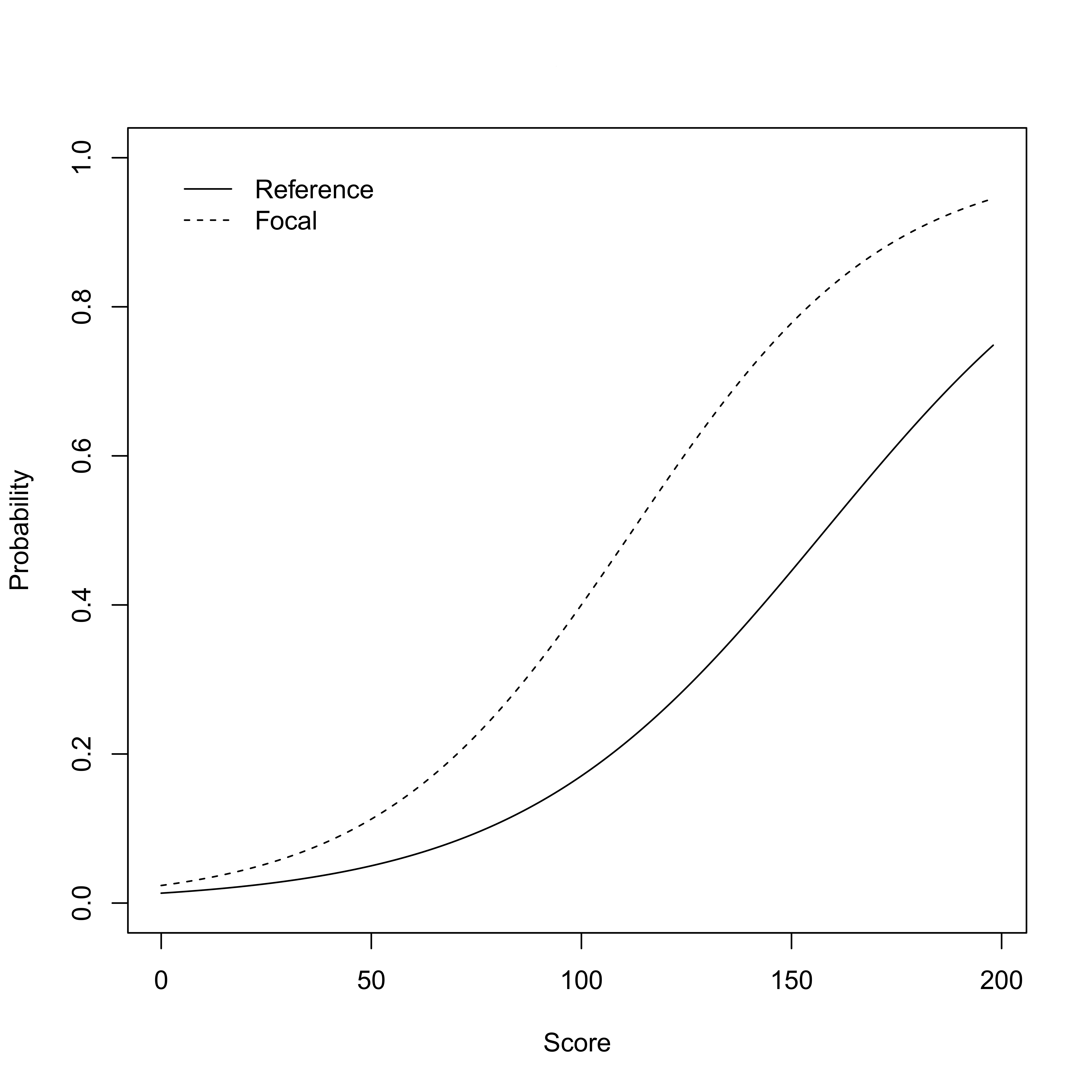

Supplement: Supplementary file 1 — High resolution images of the plots contained in the paper. (ZIP 2058 kb) [file 12909_2018_1143_MOESM1_ESM.zip › Q7FINALR2.jpg]

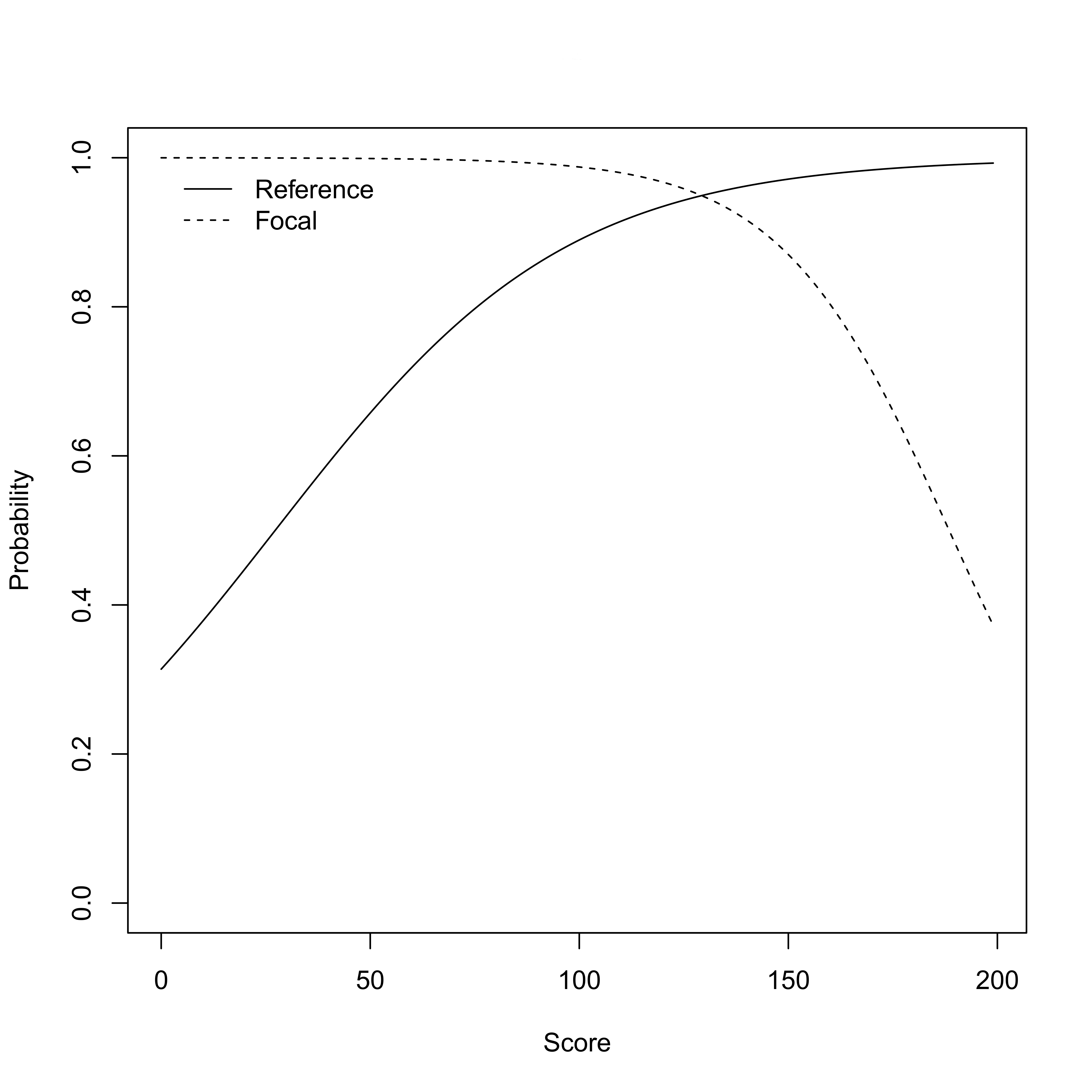

Supplement: Supplementary file 1 — High resolution images of the plots contained in the paper. (ZIP 2058 kb) [file 12909_2018_1143_MOESM1_ESM.zip › Q8FINALR2.jpg]
